# 1,1′-Dimethyl-4,4′-[(2,4-diphenyl­cyclo­butane-1,3-di­yl)dipyridinium–(*E*)-1-methyl-4-styrylpyridinium–benzene­sulfonate (0.15/1.70/2)

**DOI:** 10.1107/S1600536809034588

**Published:** 2009-09-05

**Authors:** Hoong-Kun Fun, Chanasuk Surasit, Kullapa Chanawanno, Suchada Chantrapromma

**Affiliations:** a X-ray Crystallography Unit, School of Physics, Universiti Sains Malaysia, 11800 USM, Penang, Malaysia; b Crystal Materials Research Unit, Department of Chemistry, Faculty of Science, Prince of Songkla University, Hat-Yai, Songkhla 90112, Thailand

## Abstract

In the title compound, 1.70C_14_H_14_N^+^·0.15C_28_H_28_N_2_^2+^·2C_6_H_5_O_3_S^−^, the monocation exists in the *E* configuration with respect to the ethenyl C=C double bond and is close to planar, the dihedral angle between the pyridinium and phenyl ring being 5.20 (13)°. The dication lies about an inversion centre. In the crystal, the dication occupies almost the same site occupied by monocations at (*x*, *y*, *z*) and (2 − *x*, 1 − *y*, 1 − *z*). The O atoms of the anion are disordered over two positions with occupancies of 0.75 and 0.25. In the crystal, the cations are stacked in an anti­parallel manner along the *a* axis, whereas the anions are linked into chains along the same direction by C—H⋯O hydrogen bonds. In addition, C—H⋯π and π–π inter­actions [centroid–centroid distance = 3.593 (9) or 3.6705 (16) Å] are observed.

## Related literature

The title compound was synthesized in as part of our search for non-linear optical materials. For background to non-linear optical materials, see: Lin *et al.* (2002 ▶). For related structures, see: Chanawanno *et al.* (2008 ▶); Chantrapromma *et al.* (2009*a* ▶,*b* ▶); Fun *et al.* (2009*a* ▶,*b* ▶). For the stability of the temperature controller used in the data collection, see: Cosier & Glazer (1986 ▶).
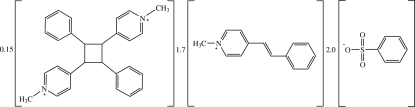


## Experimental

### 

#### Crystal data

1.70C_14_H_14_N^+^·0.15C_28_H_28_N_2_^2+^·2C_6_H_5_O_3_S^−^
*M* *_r_* = 353.43
Triclinic, 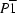

*a* = 8.4037 (1) Å
*b* = 9.8505 (1) Å
*c* = 11.0869 (1) Å
α = 68.677 (1)°
β = 88.968 (1)°
γ = 86.134 (1)°
*V* = 852.99 (2) Å^3^
*Z* = 2
Mo *K*α radiation
μ = 0.21 mm^−1^
*T* = 100 K
0.36 × 0.19 × 0.09 mm

#### Data collection

Bruker APEXII CCD area-detector diffractometer
Absorption correction: multi-scan (*SADABS*; Bruker, 2005 ▶) *T*_min_ = 0.930, *T*_max_ = 0.982
19913 measured reflections
4962 independent reflections
4045 reflections with *I* > 2σ(*I*)
*R*_int_ = 0.025

#### Refinement

*R*[*F* ^2^ > 2σ(*F* ^2^)] = 0.046
*wR*(*F* ^2^) = 0.120
*S* = 1.03
4962 reflections
387 parameters
165 restraints
H-atom parameters constrained
Δρ_max_ = 0.50 e Å^−3^
Δρ_min_ = −0.46 e Å^−3^

### 

Data collection: *APEX2* (Bruker, 2005 ▶); cell refinement: *SAINT* (Bruker, 2005 ▶); data reduction: *SAINT*; program(s) used to solve structure: *SHELXTL* (Sheldrick, 2008 ▶); program(s) used to refine structure: *SHELXTL*; molecular graphics: *SHELXTL*; software used to prepare material for publication: *SHELXTL* and *PLATON* (Spek, 2009 ▶).

## Supplementary Material

Crystal structure: contains datablocks global, I. DOI: 10.1107/S1600536809034588/ci2884sup1.cif

Structure factors: contains datablocks I. DOI: 10.1107/S1600536809034588/ci2884Isup2.hkl

Additional supplementary materials:  crystallographic information; 3D view; checkCIF report

## Figures and Tables

**Table 1:** Hydrogen-bond geometry (Å, °)

*D*—H⋯*A*	*D*—H	H⋯*A*	*D*⋯*A*	*D*—H⋯*A*
C4—H4⋯O3^i^	0.96	2.41	3.361 (2)	169
C10—H10⋯*Cg*1	0.96	2.72	3.617 (3)	157
C16—H16⋯*Cg*1^ii^	0.96	2.93	3.787 (3)	149
C20—H20*B*⋯*Cg*2^iii^	0.96	2.86	3.588 (3)	134
C20—H20*B*⋯*Cg*3^iii^	0.96	2.65	3.343 (7)	130
C10*A*—H10*A*⋯*Cg*1	0.96	2.70	3.537 (18)	146

Symmetry codes: (i) 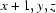; (ii) 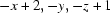; (iii) 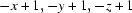. *Cg*1, *Cg*2 and *Cg*3 are centroids of the C1–C6, C14–C19 and C14*A*–C19*A* rings, respectively.

**Fig. 1.**
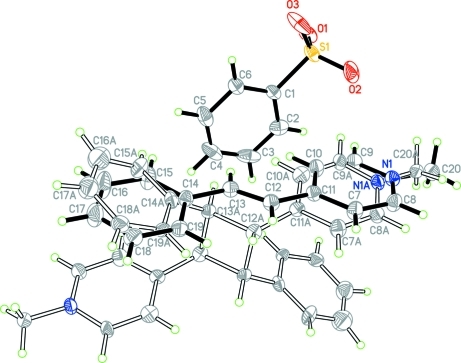
The molecular structure of the title compound, with 50% probability displacement ellipsoids and the atom-numbering scheme. The dication is shown in open bonds. Unlabelled atoms in the dication are related to the labelled atoms by the symmetry operation (2-x, 1-y, 1-z). The monocation and dication have fractional occupancies of 0.85 and 0.15, respectively. Only the minor component of the anion is shown.

**Fig. 2.**
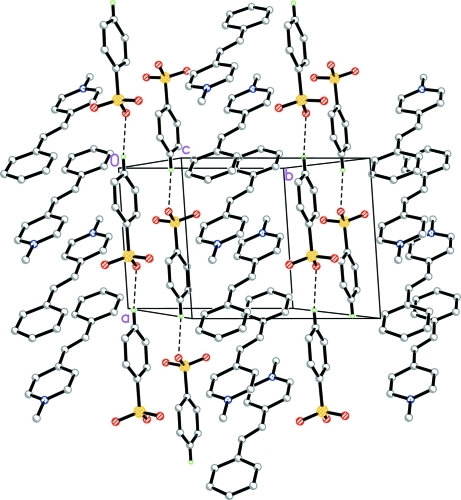
The crystal packing of the title compound viewed approximately down the c axis. H atoms not involved in hydrogen bonds (dashed lines) have been omitted. The monocation with a fractional occupancy of 0.85 is shown while the dication with an occupancy of 0.15 and minor component of the anion have been omitted for clarity.

## supplementary crystallographic information

### Comment

Recently, there is considerable interest in the synthesis of new materials with large second-order optical nonlinearities. Such materials require both molecular hyperpolarizability and orientation in a noncentrosymmetric arrangement (Lin *et al.*, 2002). Taking advantage of our previous experience in the crystal growth of some pyridinium derivatives (Chanawanno *et al.*, 2008; Chantrapromma *et al.*, 2009*a*,*b*; Fun *et al.*, 2009*a*,*b*), we report the synthesis and crystal structure of the title compound in which the dication is resulted from the unexpected [2+2] cycloaddition of the monocation. The title compound crystallizes in the centrosymmetric space group P1 and does not exhibit second-order nonlinear optical properties.

Fig. 1 shows the molecular structure of the title compound which consists of 1.70 of C_14_H_14_N^+^ cation, 0.15 of  C_28_H_28_N_2_^2+^ dication and two C_6_H_5_O_3_S^-^ anions. The monocation exists in the *E* configuration with respect to the C12═C13 double bond (C11—C12—C13—C14 = 179.2 (2)°) and is almost planar with the dihedral angle between pyridinium and C14–C19 phenyl ring being 5.20 (13)°. The dication lies on an inversion center. The dihedral angle between the pyridinium and C14A–C19A ring being 29.3 (7)°, with the C11A–C12A–C13A–C14A torsion angle being -122.2 (10)°. The O atoms of the anion are disordered over two positions with a site-occupancy ratio of 0.75:0.25. It is almost perpendicular to the monocation, with the dihedral angles between the mean plane through C1–C6 with N1/C7–C11 and C14–C19 being 87.56 (11) and 82.36 (12)°, respectively. Bond lengths are comparable with those in related structures (Chantrapromma *et al.*, 2009*a*,*b*; Fun *et al.*, 2009*a*,*b*).

In the crystal packing, the cations are stacked in an antiparallel manner along the *a* axis whereas the anions are linked into chains along the same direction by C—H···O hydrogen bonds. Weak C—H···π (Table 1) and π–π interactions [Cg4···Cg3^v^ = 3.593 (9) Å and Cg5···Cg2^v^ = 3.6705 (16) Å; symmetry code:  (v) 2-x, 1-y, 1-z; Cg1, Cg2, Cg3, Cg4 and Cg5 are centroids of the C1–C6, C14–C19, C14A-C19A, N1A/C7A–C11A and N1/C7–C11 rings, respectively] are also observed.

### Experimental

Silver(I) benzenesulfonate (compound A) was prepared by mixing a solution of benzenesulfonic acid (1.34 g, 8.5 mmol) in hot methanol (50 ml) with a solution of sodium hydroxide (0.34 g, 8.5 mmol) in methanol (30 ml), followed by addition of a solution of silver nitrate (1.44 g, 8.5 mmol) in methanol (30 ml). A colourless solution together with black solid of AgI was obtained which was then filtered. The colorless solid of compound A was collected after allowing the filtrate to stand in air for a few days. (*E*)-1-Methyl-4-styrylpyridinium iodide (compound B) was prepared by mixing 1:1:1 molar ratio solutions of 1,4-dimethylpyridinium iodide (2 g, 8.5 mmol), benzaldehyde (0.86 ml, 8.5 mmol) and piperidine (0.84 ml, 8.5 mmol) in methanol (40 ml). The resulting solution was refluxed for 3 h under a nitrogen atmosphere. The yellow solid compound formed was filtered and washed with diethylether. The title compound was prepared by mixing the solution of compound A (2.24 g, 8.5 mmol) in methanol (100 ml) and compound B (2.75 g, 8.5 mmol) in methanol (100 ml) and stirred for 30 minutes. The precipitate of silver iodide which formed was filtered and the filtrate was evaporated to give a pale orange solid product. The pale orange solid was repeatedly recrystallized for several times by dissolving the solid in hot methanol (around 323 K) to get a clear solution. The [2+2] cycloaddition of the (*E*)-1-methyl-4-styrylpyridinium occurred upon heating. Pale orange needle-shaped single crystals of the title compound suitable for X-ray structure determination were recrystallized from methanol by slow evaporation at room temperature over a few weeks (m.p. 493-495 K).

### Refinement

The fractional occupancies of the monocation and dication were initially refined to 0.847 (3) and 0.153 (3), respectively, and later for charge-balance they were fixed at 0.85 and 0.15. The sulfonate O atoms are disordered over two positions and their occupancies were initially refined to 0.758 (8) and 0.242 (8) and later fixed at 0.75 and 0.25. The displacement parameters of all atoms of the dication and sulfonate O atoms were restrained to approximate isotropic behaviour. S—O distances in the major and minor conformers of the anion were restrained to be equal. Similarity restraints were applied to equivalent interatomic distances and angles of the mono and dications. All H atoms were positioned geometrically and allowed to ride on their parent atoms, with C-H = 0.93-0.98 Å. The *U*_iso_ values were constrained to be 1.5*U*_eq_ of the carrier atom for methyl H atoms and 1.2*U*_eq_(C) for the remaining H atoms.

### Crystal data

**Table:**

1.70C_14_H_14_N^+^·0.15C_28_H_28_N_2_^2+^·2C_6_H_5_O_3_S^−^	*Z* = 2
*M**_r_* = 353.43	*F*(000) = 372
Triclinic, *P*1	*D*_x_ = 1.376 Mg m^−^^3^
Hall symbol: -P 1	Melting point = 493–495 K
*a* = 8.4037 (1) Å	Mo *K*α radiation, λ = 0.71073 Å
*b* = 9.8505 (1) Å	Cell parameters from 4962 reflections
*c* = 11.0869 (1) Å	θ = 2.2–30.0°
α = 68.677 (1)°	µ = 0.21 mm^−^^1^
β = 88.968 (1)°	*T* = 100 K
γ = 86.134 (1)°	Needle, pale orange
*V* = 852.99 (2) Å^3^	0.36 × 0.19 × 0.09 mm

### Data collection

**Table:**

Bruker APEXII CCD area-detector diffractometer	4962 independent reflections
Radiation source: sealed tube	4045 reflections with *I* > 2σ(*I*)
graphite	*R*_int_ = 0.025
φ and ω scans	θ_max_ = 30.0°, θ_min_ = 2.2°
Absorption correction: multi-scan (*SADABS*; Bruker, 2005)	*h* = −11→11
*T*_min_ = 0.930, *T*_max_ = 0.982	*k* = −13→13
19913 measured reflections	*l* = −15→15

### Refinement

**Table:**

Refinement on *F*^2^	Primary atom site location: structure-invariant direct methods
Least-squares matrix: full	Secondary atom site location: difference Fourier map
*R*[*F*^2^ > 2σ(*F*^2^)] = 0.046	Hydrogen site location: inferred from neighbouring sites
*wR*(*F*^2^) = 0.120	H-atom parameters constrained
*S* = 1.03	*w* = 1/[σ^2^(*F*_o_^2^) + (0.0518*P*)^2^ + 0.4388*P*] where *P* = (*F*_o_^2^ + 2*F*_c_^2^)/3
4962 reflections	(Δ/σ)_max_ = 0.001
387 parameters	Δρ_max_ = 0.50 e Å^−^^3^
165 restraints	Δρ_min_ = −0.46 e Å^−^^3^

### Special details

**Table:**

Experimental. The crystal was placed in the cold stream of an Oxford Cryosystems Cobra open-flow nitrogen cryostat (Cosier & Glazer, 1986) operating at 100.0 (1) K.
Geometry. All esds (except the esd in the dihedral angle between two l.s. planes) are estimated using the full covariance matrix. The cell esds are taken into account individually in the estimation of esds in distances, angles and torsion angles; correlations between esds in cell parameters are only used when they are defined by crystal symmetry. An approximate (isotropic) treatment of cell esds is used for estimating esds involving l.s. planes.
Refinement. Refinement of F^2^ against ALL reflections. The weighted R-factor wR and goodness of fit S are based on F^2^, conventional R-factors R are based on F, with F set to zero for negative F^2^. The threshold expression of F^2^ > 2sigma(F^2^) is used only for calculating R-factors(gt) etc. and is not relevant to the choice of reflections for refinement. R-factors based on F^2^ are statistically about twice as large as those based on F, and R- factors based on ALL data will be even larger.

### Fractional atomic coordinates and isotropic or equivalent isotropic displacement parameters (Å^2^)

**Table:**

	*x*	*y*	*z*	*U*_iso_*/*U*_eq_	Occ. (<1)
S1	0.38722 (5)	0.23951 (5)	0.21176 (4)	0.02809 (13)	
O1	0.3721 (6)	0.1603 (4)	0.1270 (3)	0.0343 (9)	0.75
O2	0.3317 (3)	0.3895 (2)	0.1626 (3)	0.0552 (7)	0.75
O3	0.3175 (2)	0.1588 (3)	0.33964 (19)	0.0479 (6)	0.75
O1A	0.362 (2)	0.1280 (10)	0.1600 (10)	0.030 (2)	0.25
O2A	0.3693 (9)	0.3811 (5)	0.1020 (6)	0.0475 (18)	0.25
O3A	0.3015 (8)	0.2458 (10)	0.3210 (6)	0.0534 (19)	0.25
C1	0.59528 (18)	0.22888 (18)	0.24520 (16)	0.0232 (3)	
C2	0.6978 (2)	0.3149 (2)	0.15391 (18)	0.0306 (4)	
H2	0.6591	0.3825	0.0716	0.037*	
C3	0.8603 (2)	0.3013 (2)	0.1834 (2)	0.0403 (5)	
H3	0.9334	0.3593	0.1215	0.048*	
C4	0.9193 (2)	0.2050 (2)	0.3008 (2)	0.0421 (5)	
H4	1.0310	0.1970	0.3208	0.050*	
C5	0.8174 (2)	0.1197 (2)	0.3909 (2)	0.0368 (4)	
H5	0.8567	0.0515	0.4728	0.044*	
C6	0.6546 (2)	0.13117 (19)	0.36360 (17)	0.0273 (3)	
H6	0.5819	0.0721	0.4251	0.033*	
N1	0.4628 (3)	0.7537 (2)	0.2108 (2)	0.0218 (4)	0.85
C7	0.5822 (3)	0.7427 (2)	0.4060 (2)	0.0268 (4)	0.85
H7	0.5987	0.7876	0.4681	0.032*	0.85
C8	0.4883 (3)	0.8152 (2)	0.2988 (2)	0.0272 (4)	0.85
H8	0.4400	0.9104	0.2865	0.033*	0.85
C9	0.5281 (3)	0.6190 (3)	0.2275 (3)	0.0261 (5)	0.85
H9	0.5083	0.5765	0.1643	0.031*	0.85
C10	0.6229 (3)	0.5432 (3)	0.3327 (3)	0.0272 (5)	0.85
H10	0.6683	0.4474	0.3436	0.033*	0.85
C11	0.6538 (3)	0.6040 (2)	0.42569 (19)	0.0223 (4)	0.85
C12	0.7539 (2)	0.5306 (2)	0.54043 (18)	0.0239 (4)	0.85
H12	0.7649	0.5800	0.5999	0.029*	0.85
C13	0.8318 (2)	0.3999 (2)	0.56868 (18)	0.0231 (4)	0.85
H13	0.8198	0.3505	0.5093	0.028*	0.85
C14	0.9343 (3)	0.3252 (3)	0.6822 (3)	0.0215 (5)	0.85
C15	0.9935 (5)	0.1826 (4)	0.7056 (3)	0.0363 (8)	0.85
H15	0.9662	0.1352	0.6477	0.044*	0.85
C16	1.0892 (4)	0.1066 (3)	0.8133 (3)	0.0474 (7)	0.85
H16	1.1286	0.0078	0.8288	0.057*	0.85
C17	1.1285 (3)	0.1741 (3)	0.8976 (2)	0.0385 (5)	0.85
H17	1.1958	0.1225	0.9713	0.046*	0.85
C18	1.0712 (3)	0.3162 (3)	0.8752 (3)	0.0281 (5)	0.85
H18	1.1004	0.3634	0.9329	0.034*	0.85
C19	0.9729 (3)	0.3915 (3)	0.7698 (3)	0.0241 (5)	0.85
H19	0.9302	0.4888	0.7564	0.029*	0.85
C20	0.3651 (3)	0.8339 (3)	0.0942 (2)	0.0270 (5)	0.85
H20A	0.3270	0.9271	0.0964	0.041*	0.85
H20B	0.2760	0.7787	0.0921	0.041*	0.85
H20C	0.4287	0.8481	0.0183	0.041*	0.85
N1A	0.5061 (15)	0.7265 (13)	0.2395 (13)	0.024 (3)	0.15
C7A	0.6724 (16)	0.7404 (12)	0.4039 (11)	0.027 (2)	0.15
H7A	0.7074	0.7971	0.4517	0.032*	0.15
C8A	0.5608 (16)	0.8010 (13)	0.3109 (13)	0.025 (3)	0.15
H8A	0.5184	0.8988	0.2960	0.030*	0.15
C9A	0.5571 (17)	0.5844 (14)	0.2726 (15)	0.023 (3)	0.15
H9A	0.5077	0.5263	0.2325	0.027*	0.15
C10A	0.6715 (19)	0.5233 (17)	0.3631 (16)	0.032 (4)	0.15
H10A	0.7116	0.4250	0.3774	0.038*	0.15
C11A	0.7364 (15)	0.5981 (10)	0.4331 (10)	0.020 (2)	0.15
C12A	0.8830 (9)	0.5488 (8)	0.5165 (7)	0.0196 (18)	0.15
H12A	0.8685	0.5734	0.5942	0.023*	0.15
C13A	0.9561 (9)	0.3890 (8)	0.5544 (7)	0.0198 (19)	0.15
H13A	0.9026	0.3369	0.5081	0.024*	0.15
C14A	0.9758 (18)	0.2928 (16)	0.6965 (15)	0.021 (4)	0.15
C15A	0.982 (3)	0.1440 (16)	0.7350 (16)	0.027 (4)	0.15
H15A	0.9775	0.1021	0.6694	0.032*	0.15
C16A	1.017 (2)	0.0508 (17)	0.8616 (14)	0.046 (4)	0.15
H16A	1.0048	−0.0523	0.8893	0.055*	0.15
C17A	1.0613 (17)	0.1138 (14)	0.9484 (13)	0.038 (3)	0.15
H17A	1.0972	0.0537	1.0342	0.046*	0.15
C18A	1.0605 (19)	0.2637 (15)	0.9108 (14)	0.027 (3)	0.15
H18A	1.0929	0.3049	0.9721	0.033*	0.15
C19A	1.0153 (17)	0.3559 (15)	0.7900 (14)	0.019 (3)	0.15
H19A	1.0087	0.4600	0.7667	0.023*	0.15
C20A	0.3969 (16)	0.7947 (15)	0.1336 (14)	0.021 (3)	0.15
H20D	0.3415	0.8783	0.1438	0.032*	0.15
H20E	0.3212	0.7264	0.1323	0.032*	0.15
H20F	0.4547	0.8251	0.0537	0.032*	0.15

### Atomic displacement parameters (Å^2^)

**Table:**

	*U*^11^	*U*^22^	*U*^33^	*U*^12^	*U*^13^	*U*^23^
S1	0.02068 (19)	0.0370 (2)	0.0368 (2)	0.00345 (15)	−0.00828 (16)	−0.02605 (19)
O1	0.0272 (13)	0.0477 (18)	0.0420 (19)	−0.0018 (16)	−0.0071 (15)	−0.0326 (18)
O2	0.0356 (12)	0.0368 (11)	0.106 (2)	0.0157 (8)	−0.0242 (13)	−0.0432 (13)
O3	0.0190 (9)	0.0943 (18)	0.0353 (11)	−0.0045 (11)	−0.0001 (7)	−0.0294 (12)
O1A	0.033 (3)	0.023 (3)	0.037 (4)	−0.009 (3)	−0.004 (4)	−0.013 (3)
O2A	0.044 (4)	0.025 (3)	0.077 (4)	0.010 (2)	−0.027 (3)	−0.024 (3)
O3A	0.034 (3)	0.095 (4)	0.055 (4)	0.007 (3)	−0.005 (3)	−0.057 (4)
C1	0.0198 (7)	0.0268 (7)	0.0316 (8)	0.0003 (5)	−0.0027 (6)	−0.0211 (7)
C2	0.0381 (9)	0.0303 (9)	0.0331 (9)	−0.0070 (7)	0.0059 (7)	−0.0225 (7)
C3	0.0332 (9)	0.0466 (11)	0.0616 (13)	−0.0180 (8)	0.0200 (9)	−0.0424 (11)
C4	0.0207 (8)	0.0537 (12)	0.0730 (15)	0.0012 (8)	−0.0049 (9)	−0.0486 (12)
C5	0.0316 (9)	0.0407 (10)	0.0492 (11)	0.0103 (8)	−0.0159 (8)	−0.0309 (9)
C6	0.0253 (8)	0.0295 (8)	0.0333 (9)	0.0009 (6)	−0.0033 (7)	−0.0192 (7)
N1	0.0221 (10)	0.0240 (10)	0.0210 (11)	0.0002 (8)	−0.0028 (9)	−0.0105 (9)
C7	0.0339 (12)	0.0246 (9)	0.0262 (11)	0.0034 (8)	−0.0077 (9)	−0.0149 (8)
C8	0.0339 (12)	0.0224 (10)	0.0281 (11)	0.0034 (9)	−0.0082 (10)	−0.0130 (8)
C9	0.0301 (11)	0.0274 (12)	0.0252 (12)	0.0019 (9)	−0.0047 (10)	−0.0152 (10)
C10	0.0313 (13)	0.0250 (11)	0.0304 (13)	0.0037 (10)	−0.0049 (11)	−0.0168 (10)
C11	0.0218 (9)	0.0237 (9)	0.0230 (9)	0.0005 (7)	−0.0020 (8)	−0.0108 (7)
C12	0.0258 (9)	0.0251 (9)	0.0238 (9)	−0.0006 (7)	−0.0024 (7)	−0.0125 (7)
C13	0.0257 (9)	0.0240 (9)	0.0222 (9)	−0.0005 (7)	−0.0015 (7)	−0.0115 (7)
C14	0.0232 (13)	0.0210 (11)	0.0202 (11)	0.0000 (10)	−0.0010 (9)	−0.0076 (9)
C15	0.0510 (19)	0.0297 (17)	0.0318 (16)	0.0117 (14)	−0.0175 (15)	−0.0170 (14)
C16	0.0700 (19)	0.0296 (12)	0.0459 (15)	0.0200 (12)	−0.0285 (14)	−0.0202 (11)
C17	0.0519 (14)	0.0311 (11)	0.0307 (12)	0.0068 (11)	−0.0183 (11)	−0.0099 (10)
C18	0.0368 (12)	0.0264 (13)	0.0231 (13)	−0.0054 (11)	−0.0040 (10)	−0.0105 (10)
C19	0.0260 (13)	0.0221 (12)	0.0250 (11)	−0.0014 (10)	−0.0030 (10)	−0.0095 (9)
C20	0.0292 (12)	0.0302 (13)	0.0203 (11)	0.0006 (10)	−0.0077 (9)	−0.0078 (10)
N1A	0.018 (5)	0.024 (5)	0.022 (5)	0.000 (4)	−0.004 (4)	−0.001 (4)
C7A	0.028 (4)	0.028 (4)	0.026 (4)	0.003 (4)	−0.001 (4)	−0.012 (3)
C8A	0.025 (4)	0.022 (4)	0.034 (5)	0.004 (4)	−0.007 (4)	−0.017 (4)
C9A	0.027 (5)	0.023 (5)	0.020 (5)	0.003 (4)	−0.012 (4)	−0.010 (4)
C10A	0.032 (6)	0.027 (5)	0.035 (6)	−0.003 (4)	−0.003 (4)	−0.009 (4)
C11A	0.023 (4)	0.019 (4)	0.025 (4)	0.000 (3)	−0.001 (4)	−0.017 (3)
C12A	0.025 (4)	0.021 (4)	0.016 (4)	0.001 (3)	0.004 (3)	−0.012 (3)
C13A	0.024 (4)	0.017 (3)	0.021 (4)	0.000 (3)	−0.003 (3)	−0.010 (3)
C14A	0.017 (5)	0.025 (6)	0.017 (5)	0.002 (4)	−0.003 (4)	−0.004 (4)
C15A	0.031 (5)	0.019 (6)	0.031 (6)	0.007 (4)	0.011 (4)	−0.012 (4)
C16A	0.049 (6)	0.042 (5)	0.046 (6)	−0.003 (4)	−0.001 (4)	−0.014 (4)
C17A	0.037 (5)	0.035 (5)	0.037 (5)	0.000 (4)	0.001 (4)	−0.006 (4)
C18A	0.028 (5)	0.029 (6)	0.024 (5)	−0.007 (4)	−0.004 (4)	−0.007 (4)
C19A	0.020 (5)	0.015 (5)	0.024 (5)	−0.001 (4)	0.000 (4)	−0.010 (4)
C20A	0.022 (5)	0.019 (5)	0.022 (5)	−0.001 (4)	−0.003 (4)	−0.006 (4)

### Geometric parameters (Å, °)

**Table:**

S1—O3A	1.417 (4)	C17—C18	1.384 (3)
S1—O2	1.424 (2)	C17—H17	0.96
S1—O1	1.435 (2)	C18—C19	1.386 (3)
S1—O1A	1.440 (4)	C18—H18	0.96
S1—O2A	1.481 (4)	C19—H19	0.96
S1—O3	1.483 (2)	C20—H20A	0.96
S1—C1	1.7821 (15)	C20—H20B	0.96
C1—C2	1.389 (3)	C20—H20C	0.96
C1—C6	1.392 (2)	N1A—C9A	1.353 (14)
C2—C3	1.396 (3)	N1A—C8A	1.362 (14)
C2—H2	0.96	N1A—C20A	1.434 (16)
C3—C4	1.379 (3)	C7A—C8A	1.346 (14)
C3—H3	0.96	C7A—C11A	1.391 (13)
C4—C5	1.377 (3)	C7A—H7A	0.96
C4—H4	0.96	C8A—H8A	0.96
C5—C6	1.394 (2)	C9A—C10A	1.345 (15)
C5—H5	0.96	C9A—H9A	0.96
C6—H6	0.96	C10A—C11A	1.390 (14)
N1—C8	1.348 (3)	C10A—H10A	0.96
N1—C9	1.350 (3)	C11A—C12A	1.497 (13)
N1—C20	1.476 (3)	C12A—C13A	1.5587
C7—C8	1.373 (3)	C12A—C13A^i^	1.596 (15)
C7—C11	1.398 (3)	C12A—H12A	0.98
C7—H7	0.96	C13A—C14A	1.519 (16)
C8—H8	0.96	C13A—C12A^i^	1.596 (15)
C9—C10	1.368 (3)	C13A—H13A	0.98
C9—H9	0.96	C14A—C15A	1.368 (16)
C10—C11	1.403 (3)	C14A—C19A	1.443 (15)
C10—H10	0.96	C15A—C16A	1.394 (16)
C11—C12	1.462 (3)	C15A—H15A	0.96
C12—C13	1.337 (3)	C16A—C17A	1.388 (15)
C12—H12	0.96	C16A—H16A	0.96
C13—C14	1.465 (3)	C17A—C18A	1.381 (15)
C13—H13	0.96	C17A—H17A	0.96
C14—C15	1.391 (4)	C18A—C19A	1.360 (15)
C14—C19	1.405 (3)	C18A—H18A	0.96
C15—C16	1.391 (4)	C19A—H19A	0.96
C15—H15	0.96	C20A—H20D	0.96
C16—C17	1.386 (3)	C20A—H20E	0.96
C16—H16	0.96	C20A—H20F	0.96
			
O2—S1—O1	116.5 (2)	C15—C16—H16	120.2
O3A—S1—O1A	121.0 (7)	C18—C17—C16	119.9 (2)
O3A—S1—O2A	110.9 (5)	C18—C17—H17	120.1
O1A—S1—O2A	106.6 (5)	C16—C17—H17	120.0
O2—S1—O3	112.08 (18)	C17—C18—C19	120.6 (2)
O1—S1—O3	109.9 (2)	C17—C18—H18	119.7
O2—S1—C1	108.27 (12)	C19—C18—H18	119.7
O1—S1—C1	105.4 (2)	C18—C19—C14	120.1 (2)
O3—S1—C1	103.69 (10)	C18—C19—H19	120.3
C2—C1—C6	120.17 (15)	C14—C19—H19	119.6
C2—C1—S1	120.70 (13)	C18—C19—H19A	88.2
C6—C1—S1	119.12 (13)	C14—C19—H19A	137.1
C1—C2—C3	118.75 (18)	H19—C19—H19A	46.0
C1—C2—H2	121.3	C9A—N1A—C8A	117.7 (11)
C3—C2—H2	119.9	C9A—N1A—C20A	120.6 (12)
C4—C3—C2	121.21 (19)	C8A—N1A—C20A	121.7 (11)
C4—C3—H3	118.7	C8A—C7A—C11A	121.7 (10)
C2—C3—H3	120.1	C8A—C7A—H7A	118.8
C5—C4—C3	119.89 (17)	C11A—C7A—H7A	119.5
C5—C4—H4	119.4	C7A—C8A—N1A	121.7 (11)
C3—C4—H4	120.7	C7A—C8A—H8A	118.5
C4—C5—C6	119.96 (19)	N1A—C8A—H8A	119.8
C4—C5—H5	120.9	C10A—C9A—N1A	121.0 (12)
C6—C5—H5	119.1	C10A—C9A—H9A	120.1
C1—C6—C5	120.02 (18)	N1A—C9A—H9A	118.8
C1—C6—H6	118.9	C9A—C10A—C11A	122.6 (13)
C5—C6—H6	121.1	C9A—C10A—H10A	118.2
C8—N1—C9	120.4 (2)	C11A—C10A—H10A	119.1
C8—N1—C20	120.3 (2)	C10A—C11A—C7A	114.8 (11)
C9—N1—C20	119.3 (2)	C10A—C11A—C12A	125.6 (10)
C8—C7—C11	120.58 (18)	C7A—C11A—C12A	118.6 (9)
C8—C7—H7	119.7	C11A—C12A—C13A	120.2 (4)
C11—C7—H7	119.7	C11A—C12A—C13A^i^	114.7 (8)
C8—C7—H7A	127.2	C13A—C12A—C13A^i^	90.9 (5)
C11—C7—H7A	97.8	C11A—C12A—H12A	109.9
N1—C8—C7	120.7 (2)	C13A—C12A—H12A	109.9
N1—C8—H8	119.7	C13A^i^—C12A—H12A	109.9
C7—C8—H8	119.7	C14A—C13A—C12A	119.6 (6)
N1—C9—C10	120.8 (2)	C14A—C13A—C12A^i^	114.8 (9)
N1—C9—H9	119.4	C12A—C13A—C12A^i^	89.1 (5)
C10—C9—H9	119.8	C14A—C13A—H13A	109.9
C9—C10—C11	120.5 (2)	C12A—C13A—H13A	111.0
C9—C10—H10	120.0	C12A^i^—C13A—H13A	110.9
C11—C10—H10	119.5	C15A—C14A—C19A	118.3 (13)
C7—C11—C10	117.0 (2)	C15A—C14A—C13A	120.9 (12)
C7—C11—C12	119.05 (17)	C19A—C14A—C13A	120.0 (12)
C10—C11—C12	123.97 (19)	C14A—C15A—C16A	123.1 (16)
C13—C12—C11	124.87 (17)	C14A—C15A—H15A	118.0
C13—C12—H12	117.4	C16A—C15A—H15A	118.2
C11—C12—H12	117.7	C17A—C16A—C15A	117.4 (14)
C12—C13—C14	125.76 (19)	C17A—C16A—H16A	121.1
C12—C13—H13	117.2	C15A—C16A—H16A	121.4
C14—C13—H13	117.1	C18A—C17A—C16A	120.4 (12)
C15—C14—C19	118.6 (3)	C18A—C17A—H17A	119.1
C15—C14—C13	118.9 (2)	C16A—C17A—H17A	120.5
C19—C14—C13	122.4 (2)	C19A—C18A—C17A	122.6 (13)
C14—C15—C16	121.0 (3)	C19A—C18A—H18A	118.5
C14—C15—H15	119.5	C17A—C18A—H18A	118.9
C16—C15—H15	119.5	C18A—C19A—C14A	118.0 (12)
C17—C16—C15	119.8 (2)	C18A—C19A—H19A	121.8
C17—C16—H16	120.0	C14A—C19A—H19A	120.2
			
O3A—S1—C1—C2	−134.7 (4)	C14—C15—C16—C17	−1.0 (6)
O2—S1—C1—C2	−48.72 (19)	C15—C16—C17—C18	0.7 (5)
O1—S1—C1—C2	76.6 (2)	C16—C17—C18—C19	0.7 (4)
O1A—S1—C1—C2	93.2 (5)	C17—C18—C19—C14	−1.9 (4)
O2A—S1—C1—C2	−18.1 (3)	C15—C14—C19—C18	1.6 (4)
O3—S1—C1—C2	−167.91 (17)	C13—C14—C19—C18	−179.6 (2)
O3A—S1—C1—C6	46.3 (4)	C11A—C7A—C8A—N1A	−1(2)
O2—S1—C1—C6	132.32 (18)	C9A—N1A—C8A—C7A	6(2)
O1—S1—C1—C6	−102.4 (2)	C20A—N1A—C8A—C7A	−174.9 (13)
O1A—S1—C1—C6	−85.8 (5)	C8A—N1A—C9A—C10A	−7(2)
O2A—S1—C1—C6	163.0 (3)	C20A—N1A—C9A—C10A	173.3 (15)
O3—S1—C1—C6	13.13 (18)	N1A—C9A—C10A—C11A	4(3)
C6—C1—C2—C3	0.0 (2)	C9A—C10A—C11A—C7A	1(2)
S1—C1—C2—C3	−178.99 (12)	C9A—C10A—C11A—C12A	−167.9 (13)
C1—C2—C3—C4	−0.2 (3)	C8A—C7A—C11A—C10A	−3(2)
C2—C3—C4—C5	0.3 (3)	C8A—C7A—C11A—C12A	167.1 (12)
C3—C4—C5—C6	−0.2 (3)	C10A—C11A—C12A—C13A	−13.1 (15)
C2—C1—C6—C5	0.1 (2)	C7A—C11A—C12A—C13A	178.5 (8)
S1—C1—C6—C5	179.09 (12)	C10A—C11A—C12A—C13A^i^	93.6 (15)
C4—C5—C6—C1	0.0 (3)	C7A—C11A—C12A—C13A^i^	−74.8 (13)
C9—N1—C8—C7	0.7 (4)	C11A—C12A—C13A—C14A	−122.2 (10)
C20—N1—C8—C7	−178.4 (2)	C13A^i^—C12A—C13A—C14A	118.3 (9)
C11—C7—C8—N1	0.3 (4)	C11A—C12A—C13A—C12A^i^	119.5 (9)
C8—N1—C9—C10	−1.0 (4)	C13A^i^—C12A—C13A—C12A^i^	0.0
C20—N1—C9—C10	178.2 (2)	C12A—C13A—C14A—C15A	155.5 (13)
N1—C9—C10—C11	0.3 (4)	C12A^i^—C13A—C14A—C15A	−100.4 (16)
C8—C7—C11—C10	−0.9 (3)	C12A—C13A—C14A—C19A	−34.9 (15)
C8—C7—C11—C12	179.6 (2)	C12A^i^—C13A—C14A—C19A	69.2 (15)
C9—C10—C11—C7	0.7 (4)	C19A—C14A—C15A—C16A	3(3)
C9—C10—C11—C12	−179.8 (2)	C13A—C14A—C15A—C16A	172.7 (16)
C7—C11—C12—C13	−178.3 (2)	C14A—C15A—C16A—C17A	−6(3)
C10—C11—C12—C13	2.2 (4)	C15A—C16A—C17A—C18A	4(3)
C11—C12—C13—C14	179.2 (2)	C16A—C17A—C18A—C19A	1(3)
C12—C13—C14—C15	173.1 (3)	C17A—C18A—C19A—C14A	−4(2)
C12—C13—C14—C19	−5.7 (4)	C15A—C14A—C19A—C18A	2(2)
C19—C14—C15—C16	−0.1 (6)	C13A—C14A—C19A—C18A	−168.1 (13)
C13—C14—C15—C16	−179.0 (3)		

Symmetry codes: (i) −*x*+2, −*y*+1, −*z*+1.

### Hydrogen-bond geometry (Å, °)

**Table:**

*D*—H···*A*	*D*—H	H···*A*	*D*···*A*	*D*—H···*A*
C4—H4···O3^ii^	0.96	2.41	3.361 (2)	169
C10—H10···Cg1	0.96	2.72	3.617 (3)	157
C16—H16···Cg1^iii^	0.96	2.93	3.787 (3)	149
C20—H20B···Cg2^iv^	0.96	2.86	3.588 (3)	134
C20—H20B···Cg3^iv^	0.96	2.65	3.343 (7)	130
C10A—H10A···Cg1	0.96	2.70	3.537 (18)	146

Symmetry codes: (ii) *x*+1, *y*, *z*;  (iii) −*x*+2, −*y*, −*z*+1; (iv) −*x*+1, −*y*+1, −*z*+1.
